# Induced pluripotent stem cells from domesticated ruminants and their potential for enhancing livestock production

**DOI:** 10.3389/fvets.2023.1129287

**Published:** 2023-02-20

**Authors:** Prasanna Weeratunga, Rebecca M. Harman, Gerlinde R. Van de Walle

**Affiliations:** Baker Institute for Animal Health, College of Veterinary Medicine, Cornell University, Ithaca, NY, United States

**Keywords:** ruminants, cellular reprogramming, characterization, induced pluripotency, stem cells

## Abstract

Ruminant livestock, including cattle, sheep, goat, and buffalo, are essential for global food security and serve valuable roles in sustainable agricultural systems. With the limited availability of embryonic stem cells (ESCs) from these species, ruminant induced pluripotent stem cells (iPSCs) and iPSC-like cells provide a valuable research tool for agricultural, veterinary, biomedical, and pharmaceutical applications, as well as for the prospect of translation to human medicine. iPSCs are generated by reprogramming of adult or fetal cells to an ESC-like state by ectopic expression of defined transcription factors. Despite the slow pace the field has evolved in livestock species compared to mice and humans, significant progress has been made over the past 15 years in using different cell sources and reprogramming protocols to generate iPSCs/iPSC-like cells from ruminants. This mini review summarizes the current literature related to the derivation of iPSCs/iPSC-like cells from domesticated ruminants with a focus on reprogramming protocols, characterization, associated limitations, and potential applications in ruminant basic science research and production.

## Introduction

The world population is projected to reach 9.8 billion by 2050 and 11.2 billion by 2100 (1). As a result, the demand for livestock commodities to support global food security is expected to double by 2050 (2). In both industrialized and developing agricultural systems, current livestock production practices are insufficient to fulfill projected world needs. To address this issue, the genetics of animal development, conformation, and disease resistance are being studied with the goal of improving the efficiency of animal food production. In addition, knowledge of livestock genetics has the potential to refine veterinary practices and contribute to biomedical and pharmaceutical applications that may be translatable to human medicine.

Embryonic stem cells (ESCs) are pluripotent cells typically derived from the inner cell mass of blastocyst-stage embryos (3). Ruminant ESCs can (i) provide material for genomic testing, (ii) be used to select desirable genetic traits, and (iii) be engineered to improve desirable genetic traits, each of which has the potential to expand our current knowledge of livestock genetics. Ruminant ESCs, however, are difficult to obtain and have proven hard to maintain in culture for research purposes.

Induced pluripotent stem cells (iPSCs) are pluripotent cells created by reprogramming differentiated cells. The creation of iPSCs from mouse (4), and shortly thereafter from humans (5, 6), opened new avenues for basic science research while also significantly improving the feasibility of producing and analyzing stem cells from other mammalian species. iPSCs have been produced from a wide range of eutherian mammals, including several types of domesticated ruminant species such as cattle, sheep, goats, and buffalo. With the limited availability of bona fide ESCs from these ruminants, iPSCs, which closely resemble ESCs, provide a practically limitless source of pluripotent stem cells.

The production of iPSCs from domesticated ruminants has the potential to benefit both agriculture and biotechnology. Here, we describe the methods utilized to create, characterize, and maintain, ruminant pluripotent cell lines. This mini review refers to these cell lines as iPSCs regardless of the extent to which they have been characterized (Table 1), and thus, represent authentic iPSCs. Moreover, we discuss the limitations of these cells and explore possibilities for enhancing livestock production.

**Table 1:** Summary of induced pluripotent stem cells (iPSCs) generated from cattle, sheep, goat, and buffalo.

**Reprogramming cell sources**	**Reprogramming method**	**Culture conditions**	**Pluripotency marker expression (protein, *gene*)**	**Transgene(s) detected after viral reprogramming**	**Highest reported passage**	**Differentiation**		**References**
						***In vitro***	***In vivo***	
**Cattle**								
Embryonic fibroblasts	Retroviral system; Bovine OSKMLN; Human OSKM	DMEM-F12, 20% KSR, Human FGF2, Mouse LIF; MEF feeders	ALP, SOX2, SSEA-1, SSEA-4, TRA-1-60; *OCT4, SOX2*	Yes	16	EB	Teratomas	(70)
Skin fibroblasts	Lentiviral System; Human OSKM defined-factor fusion proteins	DMEM, 15% FBS, LIF, FGF2; MEF feeders	ALP, OCT4, NANOG, SSEA1; *OCT4, KLF4, NANOG*	Yes	40	EB	Teratomas	(21)
Testicular cells	Electroporation of OCT4	DMEM, 10% FBS, Human LIF; MEF feeders	ALP, OCT4, NANOG, SOX2, SSEA-1, SSEA-4; *OCT4, SOX2, MYC, KLF4, STAT3, SUZ12, DNMT1, MEF2A*	ND	15	Ectodermal, mesodermal, and endodermal precursors	Teratomas	(20)
Mammary epithelial cells; Skin fibroblasts	Retroviral system; Mouse OSKM	DMEM, 10% FBS, LIF. BFGF; MEF feeders	ALP, OCT4, LIN28; *OCT4, KLF4, SOX2, NANOG, LIN28, REX1*	Yes	20	ND^*^	Teratomas	(61)
Fetal fibroblasts	Transposon systems; Sleeping Beauty; Mouse OSKM; PiggyBac; Human OSKMNL	DMEM-F12, 20% KSR, Human FGF2, Human LIF	ALP, OCT4, SSEA-1, SSEA-3, SSEA-4; *OCT4, KLF4, SOX2, C-MYC, NANOG, REX1*	Yes	40	ND	Teratomas	(22)
Amnion-derived cells	Transposon systems; PiggyBac; Doxycycline-inducible OSKM	MEM/F12, 20% KSR, Human FGF2, Bovine LIF, MEK/ERK inhibitor, GSK3 inhibitor, Forskolin	ALP, OCT4, NANOG; *OCT3/4, NANOG, REX1, ESRRβ, STELLA, SOCS3*	Yes	70	EB	Naïve state-like iPSCs, Contributed to ICM of blastocysts and tissues	(19)
Neural stem cells	Lentiviral system; Bovine miR-302/367	ES culture medium; MEF feeders	ALP, OCT4, NANOG; *OCT4, SOX2, NANOG*	ND	Not mentioned	ND	Teratomas	(18)
Embryonic fibroblasts	Lentiviral system; Human OSKM	DMEM/F12, 20% KSR, Human FGF2, Human LIF	ND	Yes	12	ND	ND	(71)
Embryonic fibroblasts and Wharton's jelly cells	Lentiviral system; Human OSKM/OSKMN; Retroviral system; Bovine OSKM/OSKMN	DMEM-F12, 15% KSR, Human FGF2, Human LIF; MEF feeders	ALP, SSEA1	ND	3	ND	ND	(72)
Fetal fibroblasts and adipose-derived mesenchymal cells	Lentiviral system; Human OSKM; Mouse OSKM	KO DMEM-F12, 20% KSR, Human FGF2, Mouse LIF, Human LIF	ALP, OCT4, NANOG	ND	50	EB	ND	(23)
Fetal fibroblasts	Lentiviral system; Mouse OSKM	KO DMEM-F12, 20% KSR, 5% or 20% Oxygen, Human FGF2, Mouse LIF, MEK inhibitor, GSK-3 inhibitor; MEF feeders	ALP, SOX2, OCT4; *SOX2, OCT4, STELLA*	Yes	25	EB	ND	(73)
Fetal fibroblasts	Lentiviral system; Mouse OSKM, SV40LT; Bovine Nanog	DMEM, 10% FBS, Human FGF2, Human LIF; MEF feeders	ALP, SSEA1; *OCT4, SOX2, NANOG, ESRRB, KLF4, STST3*	Yes (at passage 2); No (at passages 10 & 20)	22	EB	Teratomas	(16)
Mesenchymal stem cells	Retroviral system; Mouse OSKMLN; KDM4A	DMEM/F12 and neutral basal medium, Human FGF2, CHIR-99021, Activin A; MEF feeders	ALP, OCT4, NANOG, SOX2,; SSEA3, SSEA4, TRA-1-60; *OCT4, NANOG, SOX2*	Yes (in early passages); No (at passages 10-17)	70	EB	Naïve-like iPSCs incorporated into mouse embryos and integrated into extra-embryonic tissues	(17)
Fetal fibroblasts	Lentiviral system; Mouse OSKM	KO DMEM-F12, 20% KSR; MEF feeders	ALP, SOX2, OCT4, NANOG; *SOX2, OCT4, NANOG, ESRRβ, STELLA, LIF, OTX2*	Yes	30	EB iPSCs contributed to the ICM region of day 7 blastocysts	ND	(74)
**Sheep**					
Adult fibroblasts	Lentiviral system; Human OSKMLN, SV40LT; Tet-on inducible	DMEM/F12, 20% KSR, DOX; MEF feeders	ALP, SSEA-1, TRA-1-60, TRA-1-81, REX1, E-cadherin*; OCT4, SOX2, NANOG*	Yes	31	EB	Teratomas	(24)
Fetal fibroblasts	Lentiviral system; Mouse OSKM; Tet-on inducible	KO DMEM, 20% KSR, Human FGF2; MEF feeders	ALP, OCT4, SOX2, NANOG, SSEA-4*; SOX2, NANOG, KLF4*	Yes	20	EB	Teratomas	(25)
Embryonic fibroblasts	Retroviral system; Human OSKM	DMEM, 20% FBS, Human FGF2, Mouse LIF; MEF feeders	OCT4, NANOG; *OCT4, SOX 2*	Yes	17	EB	Teratomas Contributed to the ICM of blastocysts	(30)
Embryonic fibroblasts	Retroviral system; Mouse OSKM	KO DMEM, 20% KSR, Human FGF2, and DMEM, 15% FCS, Mouse LIF; SNL feeders	ALP, NANOG	Yes	23	EB	Teratomas Contributed to live-born chimeric lambs	(31)
Fetal fibroblasts	Retroviral system; Mouse OSKM	KO DMEM, 20% KSR, Human FGF2; MEF feeders	ALP, OCT4, FGFR2; *OCT4, SOX 2*	Yes	40	EB	SCNT to create embryos failed	(75)
Kidney cells	Lentiviral system; Human OSKMLN, SV40LT, Human TERT, p53 RNAi, ASF1A	DMEM/F12, 20% KSR, Human FGF2, Vitamin C, VPA; MEF feeders	ALP, SOX2, OCT4, NANOG, REX1, SSEA-1, TRA1-60, TRA1-81, E-Cadherin; *NANOG, OCT4, SOX2, TDGF1, DAX1, ERAS, DNMT3b, DPPA4, GDF3*	Yes (in early passages); No (by passage 53)	30	EB	Teratomas	(28)
Fibroblasts from 15-day old sheep	Transposon systems; PiggyBac; Bovine OSKM, Porcine NANOG, Human LIN28, SV40LT, Human TERT; DOX-inducible	KO DMEM, 15% FBS, Human FGF2, Human LIF, Vitamin C; STO feeders	ALP, SOX2, OCT4, NANOG; *OCT4, NANOG, SOX2, KLF4*	Yes	32	EB Chimeric contribution to early blastocysts of sheep and mice and E6.5 mouse embryos	ND	(26)
Fetal fibroblasts	Plasmid vector carrying synthetic precursor miRNAs to induce mature miR-302s/367 expression	DMEM/F12, 20% KSR, Human FGF2, Vitamin C, VPA	miR-302s/367 did not reprogram cells into iPSCs; Inhibition of proliferation and apoptosis by targeting CDK2, E2F1, E2F2, and PTEN in the cell cycle and PI3K-Akt pathways	ND	Not mentioned	NA^**^	NA	(27)
Kidney cells	Lentiviral system; Human OSKMLN, SV40LT, Human TERT; Overexpression; of miR-200c-141; Tet-on inducible	DMEM/F12, 20% KSR, Human FGF2, Vitamin C, VPA; MEF feeders	ALP, OCT4, SOX2, NANOG, REX1; *OCT4, SOX2, NANOG, DAX1*	Yes	10	EB	ND	(29)
**Goat**					
Fetal ear fibroblasts	Lentiviral system; Human OSKM	DMEM/F12, 20% KSR, Human FGF2; MEF feeders	ALP, OCT4, NANOG; *OCT4, SOX2, cMYC, NANOG, KLF4*	ND	17	EB	Teratomas	(76)
Fetal fibroblasts	Lentiviral system; Mouse OSKM; Tet-on inducible	KO DMEM, 20% KSR, Mouse LIF, Vitamin C, VPA; MEF feeders	ALP, OCT4, SOX2, NANOG, SSEA-1, TRA-1-60, TRA-1-81; *OCT4, SOX2, NANOG, KLF4, LIN28, REX1*	Yes (in early passages); No (by passage 15)	15	EB	Teratomas	(77)
Fetal fibroblasts	Lentiviral system; Bovine OSKMLN in combination with aMIR302/367cluster	DMEM/F12, 20% KSR, Mouse LIF, Human FGF2; MEF and SNL feeders	ALP, OCT4, SOX2, NANOG; *OCT4, SOX2, KLF4*	Yes	30	EB	Teratomas	(34)
Embryonic fibroblasts	Lentiviral transduction; Human OSKM, +/- PRMT5	KO DMEM, 20% KSR, Human LIF, Human FGF2; MEF feeders	ALP, OCT4, C-MYC, SSEA1, SSEA4; *SOX2, KLF4, OCT4, C-MYC, NANOG*	ND	4	EB	ND	(78)
Fetal fibroblasts	Retroviral transduction; Mouse OSKM	KO DMEM, 15% FBS, Mouse LIF, Human FGF2; MEF feeders	ALP, OCT4, NANOG, SSEA1; *OCT4, REX*	Yes	20	ND	ND	(79)
Embryonic fibroblasts	Transfection with mRNA OSKM	KO DMEM, 20% KSR, Human FGF2	ALP, OCT4, SOX2, KLF 4, NANOG, CDX2, REX, SSEA-1, TRA-1-60, TRA-1-81; *OCT4, SOX2, NANOG, DAX1, GDF3*	Yes	22	EB	ND	(32)
Ear fibroblasts	Chemical induction with small molecules CHIR98014, Forskolin, VPA, Tranylcypromine, ALK5 inhibitor, TTNPB, 3-DZnep	KO DMEM-F12, Neurobasal medium, N-2 supplement; Matrigel-treated plates MEF feeders	ALP, OCT4, SOX2; *OCT4, SOX2, NANOG, CDH1, TDGF, DAX1*	NA	Not mentioned	EB	ND	(33)
**Buffalo**					
Fetal fibroblasts	Retroviral transduction; Buffalo OSKM	DMEM, 20% FBS, Human FGF2, Human LIF; MEF feeders	ALP, OCT4, SOX2, NANOG, SSEA-1, SSEA-4, TRA-1-81, E-cadherin; *OCT4, SOX2, NANOG, LIN28*	Yes (in early passages); No (by passage 10)	10	EB	Teratomas	(37)
Fetal Fibroblasts	Chicken egg extract at various concentrations	KO DMEM/F12, 20% FBS, Human LIF, Human FGF2; BFF feeders	ALP, OCT4, NANOG, SSEA-1, TRA-1-60, TRA-1-81; *OCT4, NANOG, SOX2, KLF4, C-MYC*	NA	2	ND	ND	(36)
Fetal Fibroblasts	Lentiviral transduction; Mouse OSKM; VPA	KO DMEM/F12, 20% FBS, Human LIF, Human FGF2; BFF feeders	ALP, OCT4, NANOG, SSEA-1, TRA-1-60, TRA-1-81; *OCT4, NANOG, SOX2, KLF4, C-MYC*	Yes (in early passages); No (by passage 15)	18	EB	ND	(80)
Adipose-derived mesenchymal stem cells	Retroviral plasmids; Mouse OSKM; Hypoxic (5% O2) or normoxic; VPA	DMEM, 20% FBS, Human FGF2, Human LIF	CT4, NANOG, SSEA-4, TRA-1–81; *OCT4, NANOG*	ND	9	EB	Teratomas	(35)
Fetal Fibroblasts	Transposon systems; PiggyBac; Human SOKMNL	DMEM/F12, 20% KSR, Human LIF, Human FGF2	ALP, OCT4, NANOG, SOX 2 SSEA-1, SSEA-4, SSEA-5, TRA-1-81; *OCT4, NANOG, SOX2, KLF4, C-MYC, LIN28*	ND	15	EB	ND	(81)
Fetal Fibroblasts	Lentiviral transduction; Mouse OSKM	KO DMEM/F12, 20% FBS, Human LIF, Human FGF2; MFF feeders	ALP; *OCT4, SOX2, KLF4, c-MYC, REX1, TRA1-81*	Yes	15	EB	ND	(82)
Fetal skin fibroblasts	Transposon systems; PiggyBac; Buffalo OSKM	DMEM, 5% FBS	OCT4, SOX2; Activation of LIF, Activin, BMP4 and SMAD1/5/9	ND	Not mentioned	EB	ND	(83)

^*^ND, not done; ^**^NA, not applicable.

ALP, Alkaline Phosphate; ASF1A, Anti-Silencing Function 1A Histone Chaperone; BFF, Bovine Fetal Fibroblasts; BMP4, Bone Morphogenetic Protein 4; CDH1, Cadherin 1; CDK2, Cyclin-Dependent Kinase 2; C-MYC, MYC Proto-oncogene; DAX1, Dosage-sensitive sex reversal, adrenal hypoplasia critical region, on chromosome X gene 1; DMEM, Dulbecco's Modified Eagle Medium; DNMT1, DNA Methyltransferase 1; DNMT3B, DNA Methyltransferase 3 Beta; DOX, Doxycycline; DPPA4, Developmental Pluripotency Associated 4; E2F1, E2F Transcription Factor 2; E2F1, E2F Transcription Factor 1; EB, Embryoid bodies; ERAS, ES Cell Expressed Ras; ERK, Extracellular signal-regulated kinases; ES, Embryonic Stem; ESRRB, Estrogen-Related Receptor Beta; FGF2, Fibroblast growth factor 2; FGFR2, Fibroblast Growth Factor Receptor 2; GDF3 Gene, Growth Differentiation Factor 3; GSK3, Glycogen Synthase Kinase 3; ICM, Inner Cell Mass; KDM4A, Lysine Demethylase 4A; KLF4, Krüppel-like factor 4; LIF, Leukemia Inhibitory Factor; LIFr, Leukemia Inhibitory Factor receptor; LIN28, Lin-28 homolog A; MEF, Mouse Embryonic Fibroblasts; MEF2A, Myocyte Enhancer Factor 2A; MEK, Mitogen-Activated Protein Kinase Kinase; MEM, Minimum Essential Medium; miRNAs, microRNAs; NANOG, Homeobox protein NANOG; OCT4, Octamer-binding transcription factor 4; OSKM, OCT4, SOX2, KLF4, C-MYC; OSKMLN, OCT4, SOX2, KLF4, C-MYC, NANOG, LIN28; OTX2, Orthodenticle Homeobox 2; PRMT5, Protein Arginine Methyltransferase 5; PTEN, Phosphatase and Tensin Homolog; REX1, Reduced Expression gene 1; RNAi, RNA interference; SCNT, Somatic Cell Nuclear Transfer; SMAD, Suppressor of Mothers Against Decapentaplegic; SNL, SNL feeder cells; SOCS3, Suppressor of Cytokine Signaling 3; SOX2, SRY-Box Transcription Factor 2; SSEA-1, Stage-Specific Embryonic Antigen-1; SSEA-4, Stage-Specific Embryonic Antigen-4; STAT3, Signal Transducer and Activator of Transcription 3; STO, Sandos inbred mouse (SIM)-derived 6-thioguanine- and ouabain-resistant cells; SUZ12, SUZ12 Polycomb Repressive Complex 2 Subunit; SV40LT, SV40 large T antigen; TDGF1, Teratocarcinoma-Derived Growth Factor 1; TERT, Telomerase Reverse Transcriptase; TRA-1-60, T cell Receptor Alpha locus; TRA-1-81, Podocalyxin-Like Protein-1; VPA, Valproic acid.

## Limited availability of embryonic stem cells from domesticated ruminants

The derivation and maintenance of stable ESCs from domesticated ruminants is challenging and complicated. Over the years, there have been numerous and contradictory reports of the successful generation of ESCs from domesticated ruminants (7). However, stable, well-characterized ruminant ESCs are extremely limited in supply. The poor success rates in developing and maintaining ESCs from ruminants compared to mice or humans can be attributed to the differences in these animals' developmental processes and the need for specific culture conditions. Fundamental biological differences between species, the time point differences utilized to isolate ESCs, differences in the genes and molecular pathways that govern the pluripotency network, and poorly defined pluripotency states (naïve vs. prime) in ruminant species, may necessitate protocols designed specifically for handling ESCs from each species. In addition, the long-term culture of ruminant ESCs while maintaining full pluripotency is challenging and requires further refinements.

Nevertheless, efforts have been made to establish (putative) ruminant ESCs by applying standard or modified culture systems developed for murine and human ESCs. For instance, a culture system containing fibroblast growth factor 2 (FGF2) and an inhibitor of the canonical Wnt-signaling pathway, which was successfully used to create human ESCs, was employed to derive pluripotent cell lines from cow blastocysts with stable morphology, karyotype, pluripotency marker expression and epigenetic features (8). Likewise, ovine ESCs have been derived using similar conditions (9). The generation of caprine ESCs (10), caprine ESC-like cells (11), buffalo ESCs (12, 13) and buffalo ESC-like cells (14), has been reported as well. However, most of these cells did not maintain robust self-renewal capacity and did not develop into bona fide ESC lines capable of undergoing germline transmission.

## Generation and characterization of induced pluripotent stem cells from domesticated ruminants

Since the first reports were published in 2011, numerous studies describe the production of ruminant iPSCs and iPSC-like cells using a variety of cell sources, reprogramming systems, reprogramming factor combinations, and culture conditions. Moreover, these cultures have been characterized *in vitro* and/or *in vivo* to various degrees, and are referred to as iPSCs in this review, regardless of the extent to which they have been characterized and, thus, to what extent they represent authentic iPSCs. Table 1 illustrates variations and similarities in the generation and characterization of domesticated ruminant iPSCs across studies.

### General criteria to characterize induced pluripotent stem cells

Measuring pluripotency is a fundamental component of every stem cell-based study. Assays testing pluripotency *in vitro* include relative quantification of the expression of pluripotency genes at the mRNA level and immunocytochemistry to detect specific pluripotency markers at the protein level. Moreover, embryoid body (EB) formation assays to test the ability of the cells to form three embryonic germ-layers can be conducted. Teratoma formation in immunodeficient mice is widely used as an index of pluripotency, as it assesses the capability of the cells to differentiate into the three embryonic germ layers *in vivo*, and it provides a reliable and comprehensive validation of the functional pluripotency of the cells (15).

Descriptions of the assays used to characterize each ruminant cell line discussed are detailed in Table 1.

### Induced pluripotent stem cells from cattle

Several groups have reprogrammed bovine cells from various developmental stages and tissues, including non-conventional cell sources such as amnion-derived cells, Wharton's jelly cells, and multipotent stem cells such as neural stem cells and mesenchymal stromal cells (MSCs). These groups primarily relied on bovine, human, or murine reprogramming factors consisting of POU class 5 homeobox1 *(OCT4)*, SRY-box transcription factor 2 *(SOX)2*, KLF transcription factor 4 *(KLF4)* and MYC proto-oncogene *(c-MYC)* (OSKM) or OSKM plus Lin-28 homolog A *(LIN28)* and Nanog homeobox *(NANOG)* (OSKMNL) (Table 1). Variations such as overexpression of Lysine demethylase 4A (KDM4A) or forced expression of SV40 large T antigen, together with reprogramming factors (16, 17) or antigen reprogramming with micro RNAs (18), have also been employed. Generally, reprogramming elements were delivered via viral vectors, but the use of transposon systems encoding reprogramming factors (19) and electroporation of plasmid DNA containing a single reprogramming gene have been explored as well (20). The majority of bovine iPSCs were maintained in a dual-factor culture medium consisting of both FGF2 and leukemia inhibitory factor (LIF) for proliferation in an undifferentiated state.

Characterization of these bovine iPSCs demonstrated the expression of endogenous pluripotency factors such as ZFP42 Zinc Finger Protein *(REX1)*, Estrogen related receptor beta *(ESRRB)* and Developmental pluripotency associated 3 *(STELA)* at the transcript level, and ALP, Fucosyltransferase 4 (SSEA-1) and LIN28 at the protein level. Two studies demonstrated bovine iPSC longevity by maintaining cultures for more than 40 passages (21, 22). Both groups supplemented the culture medium with FGF2 and LIF, and one added kinase inhibitors (22). Another group reported the maintenance of bovine iPSCs for over 50 passages using FGF2 and LIF supplemented culture medium (23). In addition, bovine iPSCs cultured for over 70 passages was achieved with a culture medium containing doxycycline, histone methyltransferase, and WNT inhibitors (17, 19). Additionally, few groups demonstrated epigenetic validation of iPSCs by showing demethylation of *NANOG* and *OCT4* promoter regions in the host cells' genomes (17, 18, 21). Finally, many cultures were not subjected to experimentation to demonstrate differentiation into EBs *in vitro* and/or formation of teratomas *in vivo*, measures of functional pluripotency (Table 1). While most studies did not focus on the pluripotency state, two research groups reported the generation of bovine naïve-like iPSCs (16, 19), and both naïve state-like and primed state-like status of these cells was achieved using culture conditions for mouse and human iPSCs (17, 19).

### Induced pluripotent stem cells from sheep

Two independent groups published the first reports of ovine iPSCs in 2011 (24, 25). They used viral vectors to introduce OSKM or OSKM plus additional pluripotency genes into fibroblasts collected at different stages of development and showed that the generated iPSCs expressed multiple pluripotency markers and formed EB and teratomas. Since these two initial reports, additional groups have introduced pluripotency genes into ovine cells using a PiggyBac transposon system (26) and microRNAs (27). In addition to ovine fibroblasts, kidney cells have also been used for reprogramming (28, 29). A variety of culture media has been used across ovine iPSC studies, and different pluripotency markers have been assessed (Table 1). Most ovine iPSC cultures could form EB, some formed teratomas, and other cultures were found to contribute to early blastocysts (26, 30) and live-born chimeric lambs (31).

### Induced pluripotent stem cells from goats

All caprine iPSCs produced thus far were derived from fetal, embryonic, or adult fibroblasts. In most of the cases, lentiviral or retroviral vectors containing OSKM or OSKM plus additional pluripotency factors were utilized (Table 1). Caprine iPSCs have also been produced *via* mRNA transduction (32) and chemical induction using small molecules (33). Many different culture conditions have been used, which either included LIF or FGF2, or both. Most of the caprine iPSC cultures displayed a colony morphology like mouse iPSCs, stained positively for alkaline phosphatase (ALP), and exhibited goat-specific pluripotency markers at the transcriptional and/or protein level. Although most caprine iPSC cultures could develop into EBs, very few were able to induce teratomas *in vivo*. One study showed that directed differentiation of caprine iPSCs resulted in the *in vitro* production of trophoblast-like cells, yolk-sac endoderm-like cells and neuronal cells (34).

### Induced pluripotent stem cells from buffalo

Almost all buffalo iPSC cultures have been generated using fetal fibroblasts as the reprogramming cell source, except for one study which used adipose-derived MSCs (Table 1). In addition to viral or non-viral delivery of buffalo, mouse, or human reprogramming factors (OSKM or OSKMNL), the epigenetic modifier valproic acid has been used to enhance the reprogramming efficiency (35). Chicken egg extract added to the culture medium was also shown to be adequate to generate putative buffalo iPSCs colonies (36). Although most of these buffalo iPSC cultures were able to differentiate into EBs, only one group reported generation of *in vivo* teratomas and epigenetically validated buffalo iPSCs (35, 37).

## Limitations of ruminant induced pluripotent stem cells

Technical barriers to iPSC generation and maintenance, safety concerns when using iPSC *in vivo*, and the cost of creating and sustaining iPSC lines for therapeutic use, all contribute to the slow rate of progress in the field of iPSC research across species.

Technically, the core genes required for the establishment of pluripotency are different between mammals and are expressed at different stages of development (38). As a result, pluripotency factors other than OSKMNL need to be tested and timing of the introduction of pluripotency genes must be optimized, to determine the best methods for establishing pluripotent cell lines from ruminants. When pluripotent iPSC lines are created successfully, permanent expression of viral transgenes can interfere with differentiation into desired cell types (39). Non-viral methods of introducing pluripotency factors to target cells might circumvent this issue, but such methods have not been well-explored in ruminants to date.

For the *in vivo* use of iPSCs, safety concerns are at the forefront, primarily the risk of (i) harmful immune reactions to allogeneic cells, (ii) random integration of transduction material into the recipient's genome and (iii) differentiation of iPSCs leading to tumorigenesis. Immune reactions may be avoided by using iPSCs derived from autologous cells or altering MHC genes in iPSCs to make them less immunogenic (40). However, each of these strategies has its drawbacks. Autologous cells are not practical for large-scale, commercial treatments, and administering foreign cells that can completely avoid the host immune response introduces a risk of unchecked, inappropriate cell growth. Non-viral methods to induce pluripotency would avert the random integration of transgenes, but as mentioned above, these methods are not yet well-developed. The risk of tumor development could be reduced by differentiating iPSCs *in vitro* before administering them as a treatment (41) or by introducing a drug-inducible “suicide” gene, that can be turned on to prevent tumor growth (42). Such methods, however, are not fully optimized and currently not used *in vivo*.

The cost of generating and maintaining iPSCs for research is not trivial. The expenses required to create marketable iPSC-derived meat and other animal products in controlled laboratory environments (43), as well as those involved in commercializing iPSC-based therapies (44), are tremendous. Governments and private companies must be assured that ruminant iPSCs are a useful resource worth investing in if progress is to be made in the fields of food production, and veterinary, biomedical, and/or pharmaceutical applications.

## Potential applications of ruminant induced pluripotent stem cells for research and enhancing livestock production

**Figure 1:**
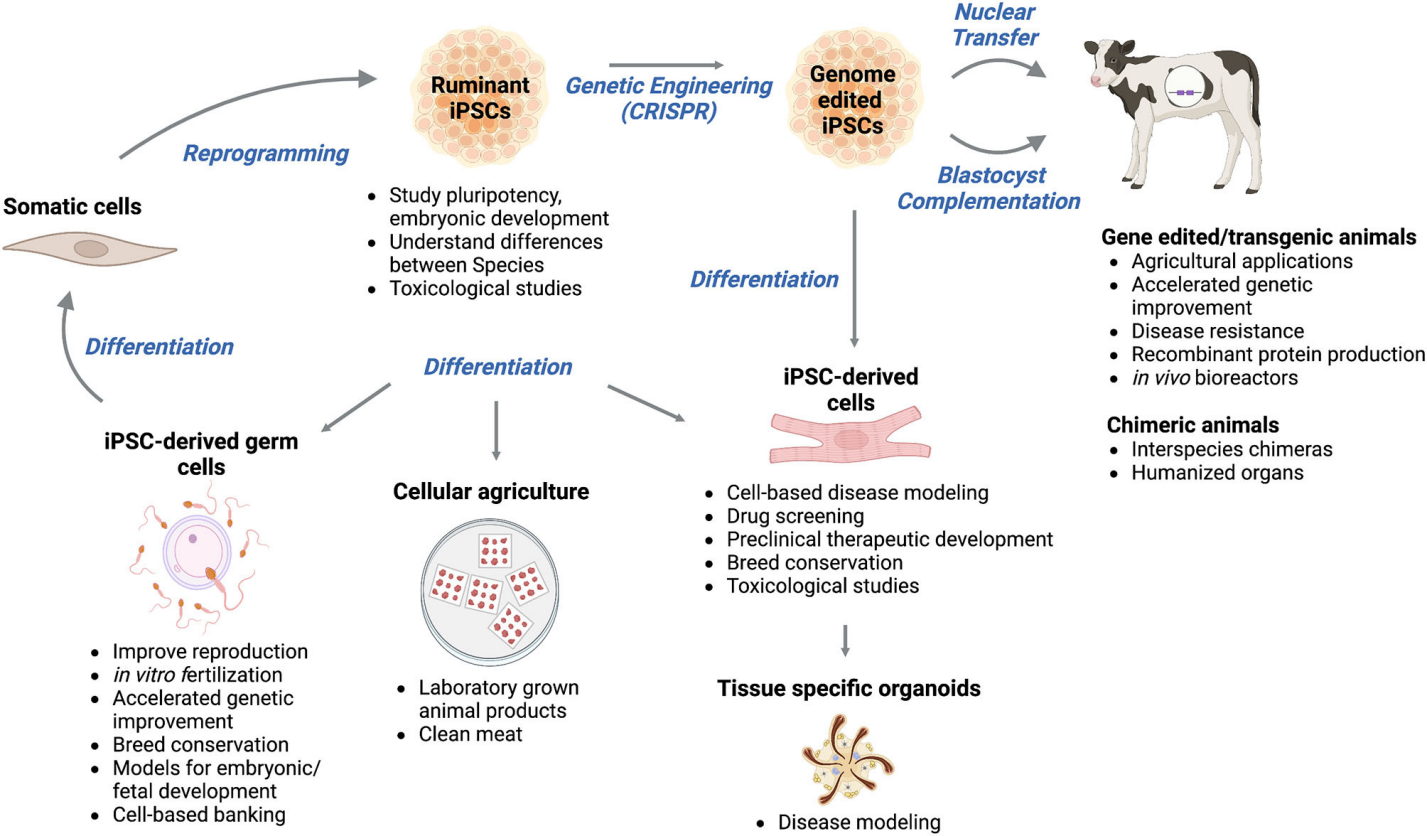
Applications and future usages of induced pluripotent stem cells (iPSCs) from ruminant livestock. Ruminant iPSCs are generated by the reprogramming of somatic cells. Ruminant iPSCs are a useful tool for studies of pluripotency, embryonic development, and understanding species differences, and can be genetically modified to create transgenic animals for various agricultural, veterinary, biomedical, and pharmaceutical applications. Ruminant iPSCs and/or gene-edited iPSCs have the potential to be used for the generation of interspecies chimeras and humanized organs. They can be the basis for disease modeling, drug screening, preclinical therapeutic development, and toxicological studies. Tissue organoids derived from ruminant iPSCs can be used to model diseases and identify effective treatment options. Ruminant iPSCs can also contribute to cellular agriculture as a source of laboratory-grown animal products. Successful generation of ruminant iPSC-derived germ cells can lead to improved reproduction, genetic improvement, breed conversation, and can be used as tools to study embryonic and/or fetal development. Figure was created using Biorender.com.

iPSCs derived from ruminant somatic cells have the potential to (i) improve agriculture, (ii) enhance veterinary, biomedical, and pharmaceutical practices, and (iii) provide knowledge that may be translatable to human medicine (Figure 1).

### Cellular agriculture

Finding alternatives to conventional farming practices is crucial, given the rising reliance on animal products for human nutrition. Compared to plant-based food sources, conventionally produced animal-based material has a larger environmental footprint, requires more soil and water, and leads to the emission of more greenhouse gases (45). Moreover, antibiotic overuse in livestock farming results in the emergence of antimicrobial-resistant bacterial strains, a significant human health concern (46). Cellular agriculture, defined as the production of animal-sourced food from cultured cells, has the potential to replace traditional farming with more environmentally friendly practices. The production of meat *in vitro* using iPSCs is proposed as a clean and prominent alternative to reduce the global burden of the livestock industry (47). In 2012, meat derived from bovine stem cells was used to create the first lab-made hamburger (48). Moreover, a commercial meat producer in the UK reported the first lab-made strips of bacon and pork belly in 2020 (49). In addition, production of cell-based seafood from fish cells and tissue cultures is also becoming popular to address the challenges associated with industrial aquaculture systems and marine capture (50). More recently, laboratory-grown meat, slaughter-free chicken, received clearance from U.S. Food and Drug Administration for human consumption (51). Additionally, generation of other iPSC-derived animal products, such as skin and fur, could reduce our dependence on industrial farming and minimize the associated environmentally harmful effects.

### Genetically modified (transgenic) ruminant livestock

The recent advancements in iPSC generation along with targeted genome editing technologies, especially the CRISPR-Cas9 system (52), have facilitated the introduction of desired genetic modifications and, combined with somatic cell nuclear transfer (SCNT) or blastocyst complementation, represent a powerful platform for transgenic animal production (53, 54).

Genetically modifying ruminants can enhance growth rates and production, improve nutrients in animal products, increase disease resistance, and enhance reproductive efficiency and fecundity. Moreover, transgenic ruminant livestock, especially those animals used for milk production such as cattle, buffalo, and goats, that are generated by genetically modified iPSCs could be used as bioreactors to produce therapeutic proteins of pharmaceutical interest. To this end, cloned transgenic cattle, which produce recombinant proteins in milk such as human coagulation factor IX has been reported (55). Additionally, transgenic ruminant livestock has the potential to significantly reduce the environmental footprint of livestock husbandry by increasing productivity and efficiency through transgenesis, which results in reduced use of land and water resources while safeguarding the soil and groundwater.

### Reproduction and conservation

Significant paradigm changes in reproduction have been made possible by prominent developments in stem cell biology. Germ cells have been derived successfully from mouse stem cells (56) and although protocols for differentiating buffalo embryonic stem cells into germ cells (57) and animal embryo-stem cell livestock laboratory breeding systems have been proposed (58), the differentiation of ruminant iPSCs to functional gametes *in vitro* has not been achieved yet.

Derivation of ruminant iPSCs may open the possibility of *in vitro* breeding. For example, selected cell lines could be differentiated to create functional gametes, which would then be used to create a new generation of embryos through *in vitro* fertilization. Such breeding schemes could substantially reduce generation intervals, enhance selection intensity, achieve more genetic gain, and preserve rare ruminant breeds and highly valuable genotypes. In addition, these cells could be expanded for the banking of genetic material and be used as donor cells for SCNT.

### Disease modeling

Ruminant diseases are widespread and have detrimental consequences on the herd and consumer health (59). The lack of appropriate ruminant disease models hampers the study of disease pathogenesis and the development of strategies to control these diseases. iPSCs are valuable tools for tissue and disease modeling, as well as preclinical therapeutic development in both human and mouse models (60). However, the use of iPSCs for ruminant disease modeling is currently limited, in part because differentiating ruminant iPSCs into clinically relevant lineages has not been well explored. One study demonstrated the potential of using iPSC technology for generating bovine mammary tissue *in vitro* (61). In this study, bovine iPSCs were successfully generated from the bovine mammary epithelium, and mammary epithelium-derived-iPSCs were differentiated back to a mammary phenotype characterized by epithelial cells expressing cytokeratin 14, cytokeratin 18, and smooth muscle actin, after treatment with progesterone (61). These studies could be complemented by generating iPSC-derived mammary organoids that can be used to explore the pathogenesis and prevention of important bovine udder diseases. Additionally, rare genetic disorders such as bovine citrullinemia and bovine leukocyte adhesion deficiency found in Holstein-Friesian cattle (62), and Chediak-Higashi syndrome found in Hereford, Brangus, and Japanese black cattle (63), may be studied using bovine iPSCs models, based on the unique advantages that iPSC cultures have shown in the modeling of rare human genetic disorders (64).

### Toxicology studies

Endocrine-disrupting chemicals (EDCs) may significantly impact the reproductive functioning of ruminant livestock, which greatly impacts agricultural production (65). Bovine iPSCs have been used to study the effects of phthalate esters, synthetic organic chemicals used in the plastic industry (20). These esters were found to significantly downregulate androgen receptors on iPSCs, which supported apoptosis (20). Ruminant iPSCs may also be used in toxicological studies investigating how pharmaceuticals, potential toxins, teratogens, and EDCs affect livestock species and humans.

### Chimera formation and growth of human organs

A composite organism of at least two genetically distinct cell populations is called a chimera. With the use of iPSC technology, the production of chimeric ruminants would allow for the genetic engineering of farm animals to improve traits of agricultural importance and the generation of biomedical models. Reports on interspecies ruminant chimeras such as sheep-goat (66) and cattle-buffalo (67) are already available. Moreover, human-ruminant chimeras could be created for use as models to study human organ development and disease pathogenesis, as well as to meet the increasing demand for and reduce the shortage of human organs. For example, human iPSCs have been engrafted in cattle pre-implantation blastocysts (68). However, enormous technical challenges and complex ethical issues must be considered and overcome before producing human organs in ruminants or any other mammals becomes feasible. The risks of human consciousness, human traits, and the creation of human gametes by such chimeras are the primary ethical concerns (69), and any attempt to create human-ruminant chimeras must be thoroughly risk-assessed, technically evaluated, and closely supervised.

## Conclusions

This mini review summarizes the work carried out to generate, maintain, and characterize iPSCs and iPSC-like cells derived from somatic cells of domesticated ruminants. Despite their undeniable potential in agriculture, conservation biology, biotechnology, and as models for preclinical research, iPSC cultures from ruminant livestock species have yet to be fully optimized. Developing uniform (i) reprogramming protocols, (ii) characterization criteria, and (iii) methods for the long-term maintenance of ruminant iPSCs needs to be prioritized to establish stable, well-defined ruminant iPSC lines that can be used to improve animal and human well-being.

## Author contributions

PW and RH: original draft preparation and editing. GV: conceptualization and editing. All authors contributed to the article and approved the submitted version.
